# A2E Induces IL-1ß Production in Retinal Pigment Epithelial Cells via the NLRP3 Inflammasome

**DOI:** 10.1371/journal.pone.0067263

**Published:** 2013-06-28

**Authors:** Owen A. Anderson, Arthur Finkelstein, David T. Shima

**Affiliations:** UCL Institute of Ophthalmology, Ocular Biology and Therapeutics, London, United Kingdom; University of Sydney, Australia

## Abstract

**Aims:**

With ageing extracellular material is deposited in Bruch’s membrane, as drusen. Lipofuscin is deposited in retinal pigment epithelial cells. Both of these changes are associated with age related macular degeneration, a disease now believed to involve chronic inflammation at the retinal-choroidal interface. We hypothesise that these molecules may act as danger signals, causing the production of inflammatory chemokines and cytokines by the retinal pigment epithelium, via activation of pattern recognition receptors.

**Methods:**

ARPE-19 cells were stimulated in vitro with the following reported components of drusen: amyloid-ß (1-42), Carboxyethylpyrrole (CEP) modified proteins (CEP-HSA), Nε-(Carboxymethyl)lysine (CML) modified proteins and aggregated vitronectin. The cells were also stimulated with the major fluorophore of lipofuscin: N-retinylidene-N-retinylethanolamine (A2E). Inflammatory chemokine and cytokine production was assessed using Multiplex assays and ELISA. The mechanistic evaluation of the NLRP3 inflammasome pathway was assessed in a stepwise fashion.

**Results:**

Of all the molecules tested only A2E induced inflammatory chemokine and cytokine production. 25 µM A2E induced the production of significantly increased levels of the chemokines IL-8, MCP-1, MCG and MIP-1α, the cytokines IL-1ß, IL-2, IL-6, and TNF-α, and the protein VEGF-A. The release of IL-1ß was studied further, and was determined to be due to NLRP3 inflammasome activation. The pathway of activation involved endocytosis of A2E, and the three inflammasome components NLRP3, ASC and activated caspase-1. Immunohistochemical staining of ABCA4 knockout mice, which show progressive accumulation of A2E levels with age, showed increased amounts of IL-1ß proximal to the retinal pigment epithelium.

**Conclusions:**

A2E has the ability to stimulate inflammatory chemokine and cytokine production by RPE cells. The pattern recognition receptor NLRP3 is involved in this process. This provides further evidence for the link between A2E, inflammation, and the pathogenesis of AMD. It also supports the recent discovery of NLRP3 inflammasome activation in AMD.

## Introduction

In the western world age related macular degeneration (AMD) is the leading cause of blindness in the elderly population. [1], [2] AMD can be classified into two groups: ‘dry’ (atrophic) and ‘wet’ (neovascular) AMD. Dry AMD accounts for approximately 90% of cases of AMD, [3] and is characterized by primary loss of the retinal pigment epithelium (RPE) with secondary atrophy of the overlying photoreceptors and underlying choriocapillaris. Vascular endothelial growth factor (VEGF) inhibitors have provided a breakthrough in the treatment of wet AMD. [4] However there is currently no effective treatment for dry AMD. Greater understanding of the pathogenesis of AMD may provide new treatment strategies for this blinding disease.

One hallmark of AMD is the presence of drusen. The deposition of extracellular material as drusen, at the level of Bruch’s membrane, precedes both forms of the disease. Drusen have been shown to contain a wide variety of substances, including amyloid-ß, advanced glycation end products (AGEs), complement components, peroxidised lipids, and vitronectin. In addition to extracellular material being deposited as drusen in Bruch’s membrane, increased amounts of insoluble lipofuscin build up within RPE cells, with increasing age. Lipofuscin has been shown to occupy 1% of the RPE’s cytoplasmic volume during the first decade of life, increasing to 19% by the age of 80 years. [5] Lipofuscin is made up of undegradable products of photoreceptor outer segment metabolism, and is the main fluorophore of the RPE. [6] A linear relationship between RPE autofluorescence and Bruch’s membrane thickness exists. [7] This implies that the ageing changes in the RPE and Bruch’s membrane are related.

Many of the molecules found in drusen are derived from the inflammatory cascade, implicating inflammation in the pathogenesis of AMD. [8] This idea was further supported following the association between complement factor H polymorphisms and AMD, [9]–[12] and histological evidence has shown the presence of macrophages near many AMD lesions (areas of Bruch’s membrane degeneration, RPE atrophy and choroidal neovascularisation (CNV)). [13]–[18] In addition aqueous humour cytokine and chemokine concentrations are elevated in patients with AMD. [19], [20].

Uncertainty exists as to whether the material, deposited in both Bruch’s membrane and the RPE, is a byproduct of disease, or actually has a pathogenic role in causing disease. Drusen and increased lipofuscin levels are found in normal ageing eyes, in the absence of disease. However components of drusen and lipofuscin have also been implicated in the pathogenesis of AMD. One theory is that molecules found in drusen and lipofuscin may act as danger signals, stimulating a range of pattern recognition receptors (PRRs) in the RPE, which in turn lead to the secretion of inflammatory chemokines and cytokines. These inflammatory chemokines and cytokines are then responsible for the recruitment and activation of immune cells (microglia, macrophages) at the retinal-choroidal interface, which leads to the chronic inflammatory process associated with AMD.

Hageman et al proposed that “the injured RPE serves as the most likely source of soluble cytokines or other stimulatory factors” that would lead to choroidal immune cell activation associated with AMD. [8] The RPE, being resident at the retinal-choroidal interface, would appear a plausible key initiator of inflammation, in response to stimulation by danger signals.

PRRs form part of the innate immune system, and their activation leads to a variety of downstream signaling effects, including cytokine secretion. They include Toll-like receptors (TLRs) and NOD-like receptors (NLRs). TLRs are membrane associated and located on both cell surface and within the cell, while NLRs are intracellular. They can be activated by exogenous pathogen-associated molecular patterns (PAMPs) as well as endogenous danger-associated molecular patterns (DAMPs), otherwise known as danger signals. Oxidative stress, lipid peroxidation and lipofuscin formation are known danger signals to RPE cells. [21] Carboxyethylpyrrole (CEP) modified proteins, found in drusen, are one such example of a danger signal. They are present in increased amounts in eyes with AMD and have been shown to be able to prime certain PRRs, known as NLRP3 (part of the intracellular NOD-like receptor family). [22] NLRP3 has also been linked to the formation of geographic atrophy, one of the hallmarks of dry AMD. [23].

Our aim was to assess whether a range of danger signals, associated with AMD, were able to induce inflammatory chemokine and cytokine production by RPE cells, and whether this occurred via the activation of PRRs. In order to achieve this aim we stimulated RPE cells in vitro and recorded chemokine and cytokine production via Multiplex assay and enzyme linked immunosorbant assay (ELISA). We then investigated the cellular pathways involved. We found that of all the molecules tested, A2E consistently and robustly stimulated chemokine and cytokine production by RPE cells. Furthermore, we found that the PRR NLRP3 was involved in this process.

Below is a brief introduction to the molecules tested:

### Amyloid-ß (1-42)

Amyloid-ß is poorly soluble peptide, prone to extracellular fibril formation. Amyloid-ß is found in drusen and has been shown to activate the complement system through blocking the function of factor I, leading to low grade chronic inflammation in sub-retinal tissues. [24] Amyloid-ß has also been shown to activate the NLRP3 inflammasome in a mouse model of Alzheimer’s disease. [25] Targeting amyloid appears to protect against RPE damage and vision loss in a model of age-related macular degeneration. [26].

### N-retinylidene-N-retinylethanolamine (A2E)

A2E, a major fluorophore in lipofuscin, is deposited in the RPE with age. It has been shown to be toxic to RPE cells in vitro, [27], [28] and is capable of oxidative damage and complement system activation. [29]–[32] Both oxidative damage and complement activation have been implicated in the pathogenesis of AMD. [9]–[12], [33], [34] Whether A2E has a direct role in causing AMD is yet unproven.

### Carboxyethylpyrrole Modified Human Serum Albumin (CEP-HSA) and CEP-Lysine

CEP protein modifications occur following oxidative damage of docosahexanoic acid (DHA), a rod outer segment lipid. CEP modified proteins, found in drusen, have been shown to stimulate neovascularisation, as well as induce a retinal changes similar to dry AMD in a mouse model. [35], [36] CEP modified proteins have been recently linked with the NLRP3 inflammasome, a multiprotein intracellular complex which is part of the innate immune response and is responsible for the production of the cytokines IL-1ß and IL-18. [37] CEP modified proteins were shown to be capable of priming the NLRP3 inflammasome, in peripheral blood derived monocytes (PBDM), further supporting involvement in the pathogenesis of AMD [22].

### Nε-(Carboxymethyl)lysine Modified Human Serum Albumin (CML-HSA)

CML modified proteins are also found in drusen. CML is an advanced glycation end product. Serum CML levels are higher in patients with AMD, as compared to age matched controls. [38] In addition CML modified proteins have been shown to promote angiogenesis in choroidal explants, as well as stimulate the production of vascular endothelial growth factor (VEGF), tumor necrosis factor alpha (TNF-α) and platelet-derived growth factor B (PDGF-B). [39].

### Vitronectin

Vitronectin is a 75 kDa glycoprotein, deposited in drusen. It is produced by RPE cells following stimulation with complement. [40] Vitronectin also forms spherical oligomers and typical amyloid fibrils. These oligomers have been shown to be toxic to RPE cells in vitro. [41].

## Materials and Methods

### Materials

All-trans-retinal, Dynasore, ethanolamine, anti ß-tubulin antibody, human serum albumin (HSA), and laminin (from Engelbreth-Holm-Swarm murine sarcoma basement membrane) were purchased from Sigma-Aldrich Ltd. 9-fluorenylmethyl ester of 4,7-dioxoheptanoic acid (DOHA-Fm) was custom manufactured by Key Organics Ltd. Lyophilised amyloid ß (1-42) and amyloid ß (42-1) were obtained from Bachem Ltd and GenScript Ltd respectively. Human vitronectin, anti-human IL-1ß antibody, and the caspase-1 inhibitor (Z-WEHD-FMK) were obtained from R&D Ltd. Anti-mouse IL-1ß antibody was obtained from Abcam Ltd. ARPE19 cells (passage 22) were obtained from American Type Culture Collection (ATCC) Ltd. Recombinant human IL-1α was obtained from Peprotech Ltd. Cathepsin-B inhibitor (CA-074 Me) and silica gel 60 were obtained from Merck Millipore Ltd, while the mouse anti-human ASC monoclonal antibody was obtained from Millipore Ltd. Short interfering RNA (siRNA), Alexa Fluor secondary antibodies, and Alexa Fluor 647 dextran were obtained from Invitrogen Ltd.

### Manufacture of Danger Signals Associated with Age Related Macular Degeneration

N-retinylidene-N-retinylethanolamine (A2E) was manufactured from all-trans-retinal and ethanolamine according to published methods. [42], [43] Purity and molecular weight were determined using high performance liquid chromatography (HPLC) and matrix-assisted laser desorption/ionization mass spectrometry (MALDI-MS) respectively (Text S1, Figure S1). The purity was 99.4%. The m/z (mass/charge) value obtained, for a single charged positive ion, was 592.5, in keeping with published results [44], [45], [46].

Carboxyethylpyrrole modified human serum albumin (CEP-HSA) was manufactured from DOHA-Fm and human serum albumin (HSA) according to published methods. [47] The degree of pyrrole modification was assessed using a pyrrole assay (Text S1). Each CEP-HSA molecule contained an average of 4.33 pyrrole groups.

Nε-(Carboxymethyl)lysine modified human serum albumin (CML-HSA) was prepared as described previously [48]. The final protein concentration was determined using the Pierce bicinchoninic acid (BCA) protein assay.

Amyloid oligomers of both the 1-42 and 42-1 versions were obtained using a protocol described by Bruban et al. [49] Amyloid ß at 500 µM (2.2572 mg/ml) in PBS (calcium and magnesium free) was incubated at 37°C for 5 days and stored at –80°C until use. Fibril formation was confirmed via thioflavin S staining. [50].

Vitronectin oligomers were obtained using a modified version of a published protocol. [41].

### Cell Culture

ARPE-19 cells were cultured in Dulbecco's Modified Eagle Medium: Nutrient mixture F-12 (DMEM/F12) (3151 mg/L D-glucose, 2.5 mM L-Glutamine, 0.5 mM sodium pyruvate, no phenol red, Invitrogen Ltd) supplemented with 10% fetal bovine serum (FBS), 100 units/ml penicillin, and 100 µg/ml streptomycin. Cultures were maintained in T75 culture flasks, at 37°C in an incubator with 5% CO_2,_ being split twice weekly. Cells were plated and treated in different ways according to the experiment being performed. Cells were not passaged beyond P35.

In order to culture cells for chemokine/cytokine release, cells were first seeded onto a 96 well flat-bottomed polystyrene tissue culture treated plate at a concentration of 30 000 cells/well (in 200 µl DMEM/F12 containing 10% FBS, 100 units/ml penicillin, 100 µg/ml streptomycin). After 24 hours the medium was replaced with 200 µl of pyruvate-free DMEM (4500 mg/L D-glucose, 4 mM L-Glutamine, no pyruvate, Invitrogen Ltd) supplemented with 1% FBS, 100 units/ml penicillin, 100 µg/ml streptomycin. The cells were cultured for a further 72 hours. Cells were then starved in 200 µl pyruvate-free DMEM containing 100 units/ml penicillin, 100 µg/ml streptomycin (no FBS). After 24 hours the cell medium was reverted to 200 µl pyruvate-free DMEM containing 1% FBS, 100 units/ml penicillin, and 100 µg/ml streptomycin for a further 48 hours. Alternatively they were pre-stimulated for 48 hours with 200 µl of pyruvate-free DMEM containing 1000 pg/ml recombinant human IL-1α, 1% FBS, 100 units/ml penicillin, 100 µg/ml streptomycin. This pre-stimulation step aimed to induce pro-IL-1ß production. [51], [52] Cells were then stimulated with various danger signals for a period of 24 hours (in 200 µl pyruvate-free DMEM containing 100 units/ml penicillin, 100 µg/ml streptomycin only). Following this period of stimulation the assay plates were centrifuged at 800 rpm for 10 minutes and the top 150 µl of the supernatant was removed from each well and frozen at −80°C. This supernatant was processed for cytokine detection using either Multiplex assay or ELISA.

In order to culture cells for siRNA knock-down, cells were first seeded at a concentration of 30 000 cells per well in 96 well cell culture plates (in 200 µl DMEM/F12 containing 10% FBS, without antibiotics). After 24 hours this medium was exchanged for 250 µl pyruvate-free DMEM (without antibiotics) supplemented with 1% FBS and 1200 pg/ml of IL-1α (to promote the manufacture of pro IL-1ß). 50 µl of transfection reagent was added. This contained 3 µl of 5 µM short interfering RNA (siRNA), 0.7 µl of Lipofectamine RNAiMax reagent (Invitrogen Ltd) and 46.3 µl of Opti-MEM-I Reduced Serum Medium (Invitrogen Ltd). This mixture was pre-incubated for 20 minutes, at room temperature, before addition to the cell culture media. The final siRNA concentration, in 300 µl of cell culture media, was 50 nM (15 pmoles). The final IL-1α concentration was 1000 pg/ml. The cells were then incubated at 37°C in 5% CO_2_ for 72 hours. Cells were then stimulated with A2E for a period of 24 hours (in 200 µl pyruvate-free DMEM containing, without FBS or antibiotics).

### Multiplex Assay and Enzyme Linked Immunosorbant Assay (ELISA)

Analysis of cell culture supernatants using a multiplex assay (eBioscience Ltd) was performed according to manufacturers instructions.

The human IL-1ß enzyme linked immunosorbant assay (ELISA) assay (Human IL-1ß DuoSet, R&D Systems Ltd) was performed according to the manufacturers instructions. In summary wells of a clear polystyrene High Bind 96 well ELISA plate (Corning Life Sciences Ltd) were loaded with mouse anti-human IL-1ß (capture antibody), followed by blocking with 1% bovine serum albumin (BSA). Cell culture supernatant was then added. Biotinylated goat anti-human IL-1ß was added to detect any IL-1ß in the cell culture supernatant, followed by streptavidin-horse radish peroxidase (S-HRP) and tetramethylbenzidine (TMB)/H_2_O_2_. The reaction was stopped with 1 M H_2_SO_4_ and absorbance was read at 450 nm using a microplate reader (Turner Biosystems Ltd).

### Immunocytochemistry

ARPE-19 cells, on 13 mm laminin-coated glass coverslips, were placed in a 24 well cell culture plate, cell side up. All steps occurred at room temperature. Cells were initially fixed with 4% paraformaldehyde for 10 minutes. The cells were then permeabilised for 3–5 minutes with 500 µl of 0.1% Triton-X-100 in PBS. Non-specific binding was then blocked for 15 minutes with 500 µl of 5% goat serum in PBS, containing 0.2% BSA. Primary and Alexa Fluor 647 secondary antibody were diluted in 5% goat serum in PBS containing 0.2% BSA. Coverslips were then mounted onto slides and viewed using a confocal fluorescent microscope (Zeiss LSM700).

### Immunohistochemistry

The ABCA4 knockout mice were a gift from Professor Gabriel Travis, Jules Stein Eye Institute, UCLA School of Medicine. The matched control group 129S2/SvHsd were obtained from Harlan Laboratories Ltd. The animals were housed in accordance with the ARVO Statement for the Use of Animals in Ophthalmic and Vision Research. Animals were aged to 35 weeks under normal conditions, eyes were removed and frozen in Tissue-Tek CRYO-OCT Compound (Andwin Scientific Ltd) over liquid nitrogen. Frozen sections were cut and stained using standard immunohistochemical techniques, as described above. The primary antibody was anti-mouse IL-1ß (Abcam Ltd) with the secondary being Alexa Fluor 647 donkey anti-rabbit IgG (Invitrogen Ltd). Rabbit IgG (Millipore Ltd) was used as a negative control.

### Western Blot

Proteins were concentrated from cell culture supernatant using methanol-chloroform precipitation at room temperature. Remaining cells were lysed with ice cold RIPA buffer (Sigma-Aldrich Ltd) containing 2× HALT protease and phosphatase inhibitor (Thermo Scientific Ltd).

Samples were separated by SDS-PAGE (15%), blotted onto nitrocellulose membrane, and incubated with anti-human IL-1ß (R&D systems) and anti ß-tubulin (Sigma-Aldrich Ltd), followed by secondary antibodies and a standard enhanced chemiluminescence (ECL) reaction (Amersham Ltd) was performed according to the manufacturer’s instructions. As a positive control for IL-1ß, we used recombinant human IL-1ß (Peprotech Ltd).

### Statistics

All figures are representative of at least 3 separate experiments. For comparison of paired data the Student’s t test was performed. For comparison of multiple sets of data one-way ANOVA, including the Bonferroni post test, was performed. Results are expressed as mean +/− standard deviation. Statistical analysis was performed using Prism 5 (GraphPad Software Ltd) with p<0.05 considered statistically significant.

## Results

### A2E Stimulated Widespread Chemokine and Cytokine Production by ARPE-19 Cells

RPE cells were cultured in the presence of a variety of danger signals (amyloid ß, A2E, CEP-HSA, CEP-Lysine, CML-HSA, and vitronectin). Cell culture supernatant was collected and processed using a Multiplex assay. The assay was designed to detect the following:

- The human chemokines IL-8, MCP-1, MIG, MIP-1α, MIP-1ß
- The human cytokines G-CSF, IFN-γ, IL-1ß, IL-2, IL-6, TNF-α
- Human VEGF-A

We found that ARPE-19 cells constitutively secreted the chemokines IL-8 and MCP-1 as well as the protein VEGF-A. None of the other chemokines or cytokines tested were present. Of the potential danger signals, only A2E induced a significant increase in the chemokines or cytokines tested. Upon incubation with A2E there was a significant rise in production of the chemokines IL-8, MCP-1, MCG and MIP-1α (all p<0.0001, one-way ANOVA). There was also a significant rise in the cytokines IL-1ß, IL-2, IL-6, and TNF-α (all p<0.0001, one-way ANOVA). VEGF-A levels also significantly increased (p<0.0001, one-way ANOVA). There was no change in the levels of MIP-1ß, G-CSF and INF-γ (Table 1).

**Table 1:** Multiplex assay of cell culture supernatant from ARPE-19 cells stimulated with various danger signals.

		Amyloid-ß	A2E	CEP-HSA	CEP-Lysine	CML-HSA	Vitronectin
Chemokine	IL-8	115.1 (62.1)	**40.4 (10.7)**	282.7 (37.6)	40.4 (10.7)	282.7 (37.6)	107.1 (41.1)
		30.6 (21.0)	**1225.5 (24.6)**	217.8 (29.1)	74.6 (22.0)	328.3 (70.7)	92.1 (14.1)
	MCP-1	3358.0 (602.1)	**1716.0 (222.3)**	5258.8 (122.0)	1716.0 (222.3)	5258.8 (122.0)	3827.9 (707.1)
		2412.4 (154.8)	**7986.7 (198.6)**	4756.9 (211.2)	2186.6 (131.9)	4872.8 (355.4)	3984.8 (214.6)
	MIG	0.0 (0.0)	**0.0 (0.0)**	0.0 (0.0)	0.0 (0.0)	0.0 (0.0)	0.0 (0.0)
		0.0 (0.0)	**48.3 (4.9)**	0.0 (0.0)	0.0 (0.0)	0.0 (0.0)	0.0 (0.0)
	MIP-1α	0.0 (0.0)	**0.0 (0.0)**	0.0 (0.0)	0.0 (0.0)	0.0 (0.0)	0.0 (0.0)
		0.0 (0.0)	**96.6 (12.5)**	0.0 (0.0)	0.0 (0.0)	0.0 (0.0)	0.0 (0.0)
	MIP-1ß	0.0 (0.0)	0.0 (0.0)	0.0 (0.0)	0.0 (0.0)	0.0 (0.0)	0.0 (0.0)
		0.0 (0.0)	0.0 (0.0)	0.0 (0.0)	0.0 (0.0)	0.0 (0.0)	0.0 (0.0)
Cytokine	G-CSF	0.0 (0.0)	0.0 (0.0)	0.0 (0.0)	0.0 (0.0)	0.0 (0.0)	0.0 (0.0)
		0.0 (0.0)	0.0 (0.0)	0.0 (0.0)	0.0 (0.0)	0.0 (0.0)	0.0 (0.0)
	IFN-γ	0.0 (0.0)	0.0 (0.0)	0.0 (0.0)	0.0 (0.0)	0.0 (0.0)	0.0 (0.0)
		0.0 (0.0)	0.0 (0.0)	0.0 (0.0)	0.0 (0.0)	0.0 (0.0)	0.0 (0.0)
	IL-1ß	0.0 (0.0)	**0.0 (0.0)**	0.0 (0.0)	0.0 (0.0)	0.0 (0.0)	0.0 (0.0)
		0.0 (0.0)	**76.5 (5.4)**	0.0 (0.0)	0.0 (0.0)	0.0 (0.0)	0.0 (0.0)
	IL-2	0.0 (0.0)	**0.0 (0.0)**	0.0 (0.0)	0.0 (0.0)	0.0 (0.0)	0.0 (0.0)
		0.0 (0.0)	**261.7 (9.8)**	0.0 (0.0)	0.0 (0.0)	0.0 (0.0)	0.0 (0.0)
	IL-6	0.0 (0.0)	**0.0 (0.0)**	0.0 (0.0)	0.0 (0.0)	0.0 (0.0)	0.0 (0.0)
		0.0 (0.0)	**21.4 (1.0)**	0.0 (0.0)	0.0 (0.0)	0.0 (0.0)	0.0 (0.0)
	TNF-α	0.0 (0.0)	**0.0 (0.0)**	0.0 (0.0)	0.0 (0.0)	0.0 (0.0)	0.0 (0.0)
		0.0 (0.0)	**46.5 (1.7)**	0.0 (0.0)	0.0 (0.0)	0.0 (0.0)	0.0 (0.0)
Other	VEGF-A	673.0 (63.7)	**458.8 (44.1)**	955.3 (40.4)	458.8 (44.1)	955.3 (40.4)	623.4 (106.6)
		485.1 (47.3)	**1169.8 (39.5)**	923.7 (32.8)	577.6 (91.5)	913.3 (62.9)	635.2 (17.9)

Cells were incubated for 24 hours with danger signals associated with AMD or the corresponding negative control. Cells were not pre stimulated with IL-1α. Cell culture supernatant was then assessed for the presence of twelve different proteins (grouped as chemokines, cytokines and other) using Multiplex assay. Concentrations of these proteins are expressed in pg/ml. Mean values (with standard deviation in brackets) are presented. The upper value represents stimulation with the negative control. The lower value represents stimulation with the molecule of interest. Each assay was performed thee times (n  = 3) using three separate cell culture wells. Statistically significant rises in chemokine/cytokine/other level are highlighted in bold (all p<0.0001, one-way ANOVA).

Test molecules: Amyloid-ß (1-42) 10 µM (negative control = Amyloid-ß (42-1)), A2E (dissolved in DMSO) 25 µM (negative control = DMSO only), CEP-HSA 1 mg/ml (negative control = HSA), CEP-Lysine (dissolved in DMSO) 100 µM (negative control = DMSO only), CML-HSA 1 mg/ml (negative control = HSA), Vitronectin 100 nM (negative control = Nil).

AMD = Age related macular degeneration, A2E = N-retinylidene-N-retinylethanolamine, CEP-HSA = Carboxyethylpyrrole modified human serum albumin, CEP-Lysine = Carboxyethylpyrrole modified lysine, CML-HSA = Nε-(Carboxymethyl)lysine modified human serum albumin, DMSO = Dimethyl sulphoxide, HSA = Human serum albumin.

### A2E Induced IL-1ß Production by ARPE-19 Cells in a Dose Dependent Manner

IL-1ß has also been shown to be one of the initial cytokines produced following PRR activation. [21], [25] IL-1ß has been shown to influence downstream production of other chemokines/cytokines by the RPE. Stimulation of RPE cells by IL-1ß induces the production of IL-6, IL-8, and MCP-1. [53], [54], [55] As we were interested in the activation of PRRs by danger signals, we therefore chose to investigate the mechanism of IL-1ß release by A2E in greater detail.

**Figure 1:**
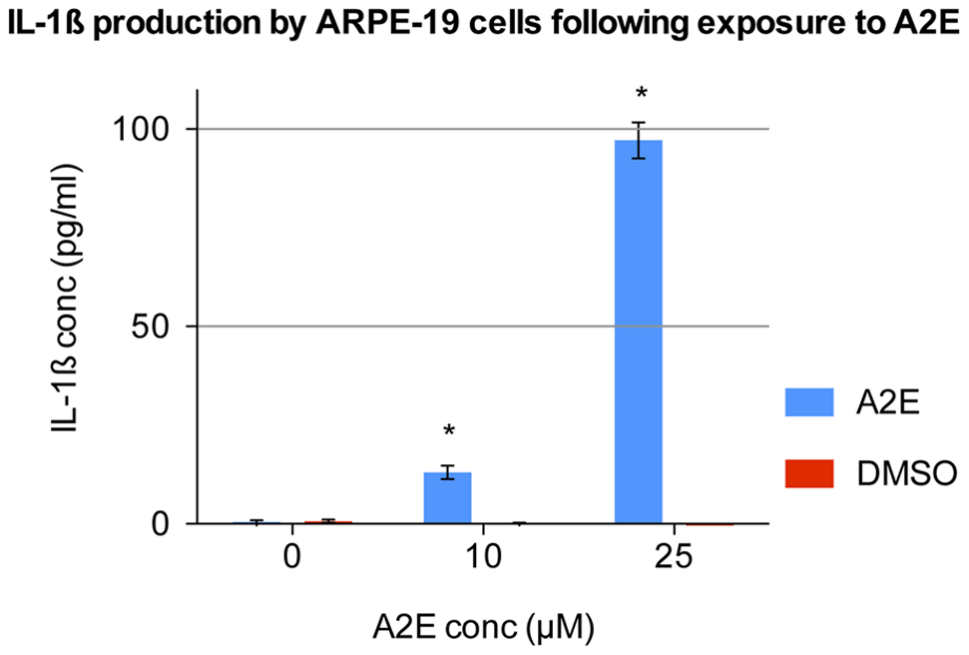
IL-1ß production by ARPE-19 cells following exposure to A2E. ARPE-19 cells were prestimulated with IL-1α and then treated with 0, 10 and 25 µM A2E for a period of 24 hours. IL-1ß levels were recorded in the supernatant via ELISA. A2E stock was dissolved in DMSO. Therefore DMSO at the same concentration, but without A2E, was used as a negative control. Four separate wells were stimulated with each concentration (n  = 4). Error bars represent standard deviation. (*) 10 and 25 µM A2E significantly increased IL-1ß production (p<0.0001, one-way ANOVA).

Pro-IL-1ß is localised intracellularly and is only released upon proteolytic cleavage to the mature form. Therefore we wished to investigate IL-1ß extracellular release further to see if PRRs were involved. First, ARPE-19 cells were incubated with different concentrations of A2E for 24 hours. A2E induced a significant increase in IL-1ß release, in a dose dependent manner (Figure 1).

**Figure 2:**
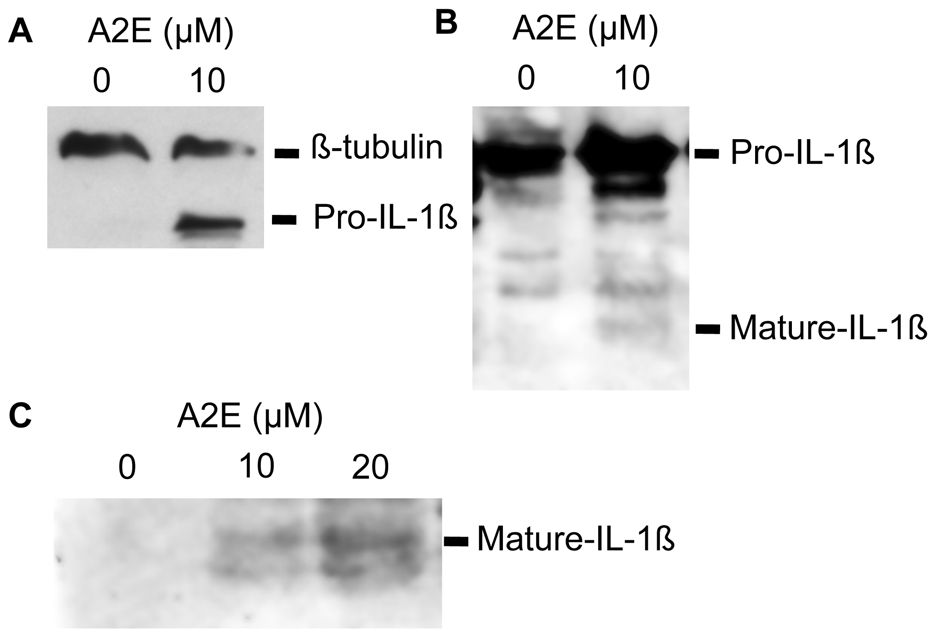
Upregulation of pro-IL-1ß and conversion to mature IL-1ß following exposure to A2E. (**A**) Western blot showing upregulation of pro-IL-1ß following stimulation with A2E. ARPE-19 cells were prestimulated with IL-1α and then treated with 0 and 10 µM A2E for a period of 24 hours. Cells were lysed (on ice in the presence of a protease and phosphatase inhibitor) and equal amounts of lysate (assessed via BCA protein assay) underwent western blotting. Membranes were probed with anti-human IL-1ß, which detects both the pro and mature form of the cytokine. Staining with ß-tubulin was used to confirm that comparable amounts of lysate were used. (**B**) Western blot of cell lysate showing both upregulation of pro-IL-1ß, with some conversion to mature IL-1ß, following stimulation with A2E. (**C**) Western blot of cell culture supernatant showing increasing amounts of mature IL-1ß in the supernatant, with increasing concentration of A2E.

This data was obtained from proliferating, undifferentiated ARPE-19 cells. To assess whether this still occurred in cells showing features more like differentiated retinal pigment epithelium, ARPE-19 cells were cultured under specific conditions to promote differentiation (Text S1, Figure S2). Transmission electron microscopy of 6-month old cells demonstrated many features seen in differentiated retinal pigment epithelial cells, including monolayer formation, intra-cytoplasmic melanin granules, apical microvilli, apical tight junctions, and zonula occludens-1 staining between adjacent cells. Stimulation of these differentiated ARPE-19 with A2E also induced IL-1ß release (Figure S3). A2E exposure had no detectable effect on IL-1ß release in microvascular endothelial cells (data not shown). Western blotting of ARPE-19 cells was used to confirm generation of mature IL-1ß. A2E exposure increased total levels of intracellular pro-IL-1ß, as well as a small but reproducible level of the mature form. Importantly, mature IL-1ß was also detected in the cell culture supernatant (Figure 2).

### A2E Stimulated IL-1ß Release is Dependent on Endocytosis

A2E has been shown to enter ARPE-19 cells via endocytosis, becoming localized in lysosomes. [56] We therefore wished to assess whether endocytosis was an important initial step in their production of IL-1ß.

**Figure 3:**
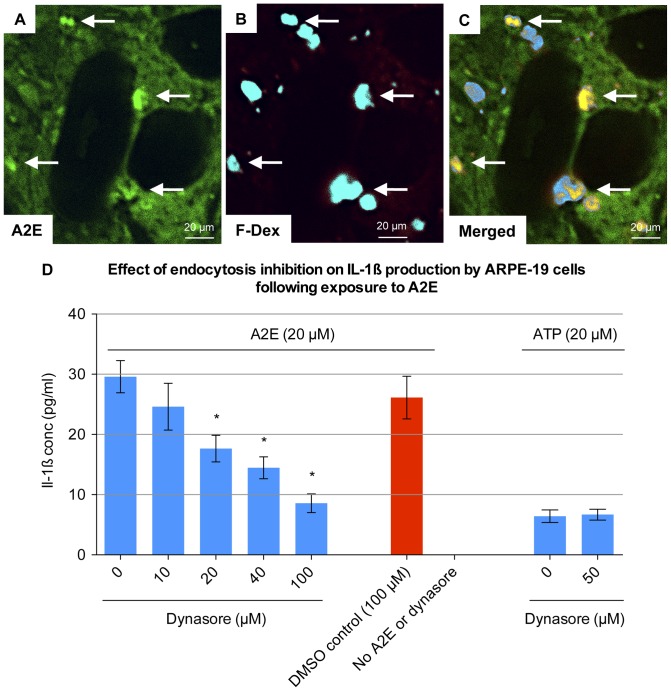
Endocytosis of A2E by ARPE-19 cells. (**A–C**) ARPE-19 cells were incubated with 20 µM A2E and 10 µM fixable 10 kDa Alexa Fluor 647 dextran for 6 hours. Cells were fixed with 4% PFA for 10 min, but not permeabilised, and viewed via confocal microscopy. Autofluorescence of A2E was viewed at an excitation frequency of 490 nm, while the Alexa Fluor dextran was viewed at an excitation frequency of 650 nm. There was no significant bleeding of A2E autofluorescence into the emission range of Alexa Fluor 647 dextran (data not shown). A2E colocalised with Alexa Fluor dextran in intracellular vesicles (arrows). (**D**) ARPE-19 cells were pre-stimulated for 48 hours with 1000 pg/ml IL-1α and then incubated with 20 µM A2E for 24 hours in the presence of 0, 10, 20, 40 and 100 µM Dynasore. Dynasore stock was dissolved in DMSO. Therefore DMSO at the same concentration, but without Dynasore, was used as a negative control. Four separate wells were stimulated with each concentration (n  = 4). IL-1ß levels were recorded in the supernatant via ELISA. Cells were also treated with 20 µM ATP in the presence of 0 and 50 µM Dynasore. Error bars represent standard deviation. (*) 20, 40 and 100 µM of Dynasore significantly inhibited IL-1ß production (p<0.0001, one-way ANOVA).

In order to confirm that A2E entered the cells via endocytosis, we incubated ARPE-19 cells with A2E in the presence of Alexa Fluor dextran. Fluorescent dextrans have been shown to label endocytic vesicles. They do not cross cell membranes or adsorb appreciably onto cell surfaces. [57] We demonstrated colocalisation of A2E with Alexa Fluor dextran, to intracellular compartments (Figure 3). This suggested that A2E entered the cell via endocytosis.

In order to confirm that endocytosis was an important initial step in the production of IL-1ß, we inhibited endocytosis using the small molecule dynamin inhibitor, Dynasore. [58] We then assessed whether this had an effect upon IL-1ß production. We found that Dynasore significantly inhibited A2E induced IL-1ß production, in a dose dependent manner (p<0.0001 for 20, 40 and 100 µM Dynasore, one-way ANOVA) (Figure 3).

**Figure 4:**
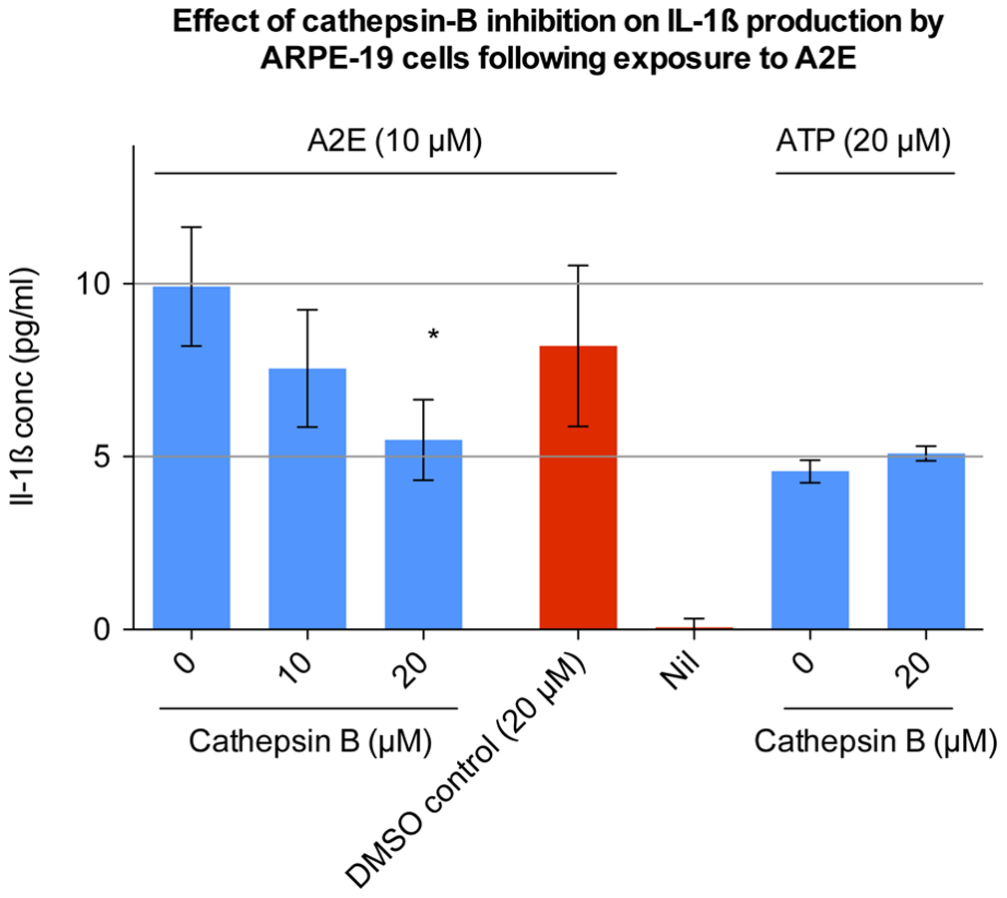
Effect of cathepsin-B inhibition on IL-1ß production by ARPE-19 cells following exposure to A2E. Undifferentiated ARPE-19 cells were pre-stimulated for 48 hours with 1000 pg/ml IL-1α. During the last 24 hours of pre-stimulation, 0, 10 or 20 µM of cathepsin-B inhibitor was added. The medium was then exchanged for serum free DMEM containing 10 µM A2E, along with the cathepsin-B inhibitor (at the same concentration as during the preceding pre-stimulation step). After 24 hours the cell culture supernatant was collected and processed using ELISA. Cathepsin-B inhibitor stock was dissolved in DMSO. Therefore DMSO at the same concentration, but without cathepsin-B inhibitor, was used as a negative control. The effect of cathepsin-B inhibition on ATP (20 µM) induced IL-1ß production was also assessed. Eight separate wells were stimulated with each concentration (n  = 8). Error bars represent standard deviation. (*) 20 µM of Cathepsin-B inhibitor significantly inhibited IL-1ß production as compared to the DMSO control (p<0.0001, one-way ANOVA).

ATP exposure was chosen as an additional control. ATP also has the ability to stimulate IL-1ß production in ARPE-19 cells. [22] The pathway of ATP induced IL-1ß production is independent of endocytosis, occurring through stimulation of cell surface P2 purinergic receptors. [59] Release of IL-1ß, by ATP, was not inhibited by Dynasore. The relationship between the endocytosis of A2E, and IL-1ß release led us to consider whether the NLRP3 inflammasome was involved. NLRP3 activation has been associated with IL-1ß production, following the endocytosis of other danger signals, including amyloid-ß, asbestos, silica, uric acid crystals, and cholesterol crystals. [25], [60], [61], [62] The endocytosis of these danger signals is believed to damage endo-lysosomes, releasing cathepsin-B, and thus activating the NLRP3 inflammasome. [25] To assess a role for cathepsin-B, ARPE-19 cells were stimulated with A2E, in the presence of different concentrations of a cathepsin-B inhibitor. Cathepsin-B inhibitor significantly inhibited A2E induced IL-1ß production (p<0.0001 for 20 µM cathepsin-B inhibitor, one-way ANOVA) (Figure 4), but did not have any effect on the release of IL-1ß following exposure to ATP. This data is consistent with a role for the inflammasome in A2E-induced IL-1ß release, but we sought further direct proof investigating the three main components of the NLRP3 inflammasome.

### A2E Altered the Cytoplasmic Localisation of ASC Protein in ARPE-19 Cells

ASC is the inflammasome adaptor protein. Along with the NOD-like receptor protein P3 (NLRP3), and activated caspase-1, it forms a multiprotein intracellular complex known as the NLRP3 inflammasome, which is responsible for the production of the cytokines IL-1ß and IL-18. [37] ASC is also known as the apoptosis-associated speck-like protein containing a carboxy-terminal CARD. Upon activation it has been shown to condense in the cytoplasm, forming large oligomers, visible on confocal microscopy. These complexes can be used as an ‘optical reporter’ of ASC activation. [63].

To assess ASC activity in the presence of A2E, ARPE-19 cells were cultured on coverslips and then incubated for 6 hours with A2E. DMSO acted as a negative control.

A masked examiner counted the number of ASC complexes present per field.

**Figure 5:**
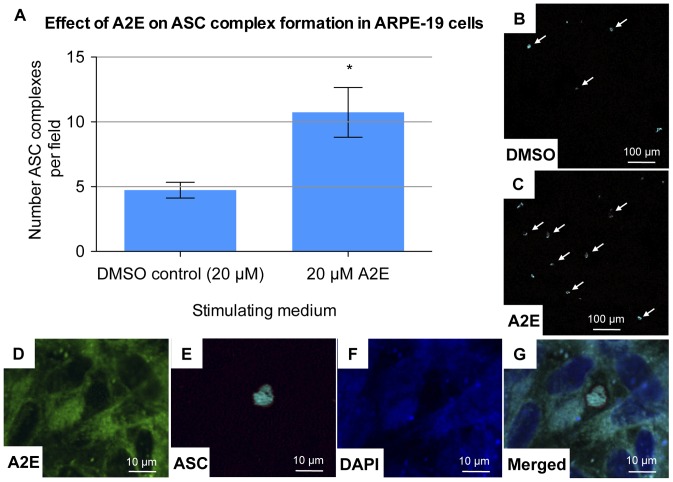
Effect of A2E on ASC complex formation in ARPE-19 cells. To assess ASC activity in the presence of A2E, ARPE-19 cells, cultured on laminin coated glass coverslips, were incubated for 6 hours with 20 µM A2E. A2E stock was dissolved in DMSO. Therefore DMSO at the same concentration, but without A2E, was used as a negative control. Coverslips were then removed and immediately stained. The primary antibody used was a mouse anti-human ASC monoclonal antibody at 1 µg/ml. An Alexa Fluor 647 goat anti-mouse IgG was used as a secondary antibody. A masked examiner counted the number of ASC complexes in five randomly assigned fields per slide. A mean count per field was then calculated for each slide. This was performed for three slides per group (n  = 3). Error bars represent standard deviation. (**A**) Number of ASC complexes per field for 20 µM A2E and for the DMSO control. (*) 20 µM of A2E significantly increased the number of ASC complexes visualized (p = 0.0067, one-way ANOVA). (**B–C**) Low magnification view of ASC complexes (some are highlighted with arrows). An increased number of complexes were seen per field, in the presence of A2E. (**D–G**) High magnification view of a cytoplasmic ASC complex (far-red). A2E shows autofluorescence in both the green and blue spectrum. Hence the DAPI nuclear stain is blurred by A2E autofluorescence. There is no bleeding of A2E autofluorescence into the ASC far-red spectrum.

There were significantly more ASC complexes in the A2E group, as compared to the DMSO (negative control) group (p = 0.007– unpaired t test) (Figure 5). This demonstrated that A2E induces alterations in ASC localization within the cell, supporting the involvement of ASC in A2E induced downstream signaling within the ARPE-19 cell.

### IL-1ß Production by A2E Stimulated ARPE-19 Cells is Dependent upon NLRP3

Having demonstrated ASC complex formation, we went on to investigate the involvement of the NOD-like receptor protein P3 (NLRP3). Specific NLRP3 siRNA knock down was performed to assess the role of the NLRP3 protein in IL-1ß production by cells stimulated with A2E. This was compared with a negative control siRNA.

**Figure 6:**
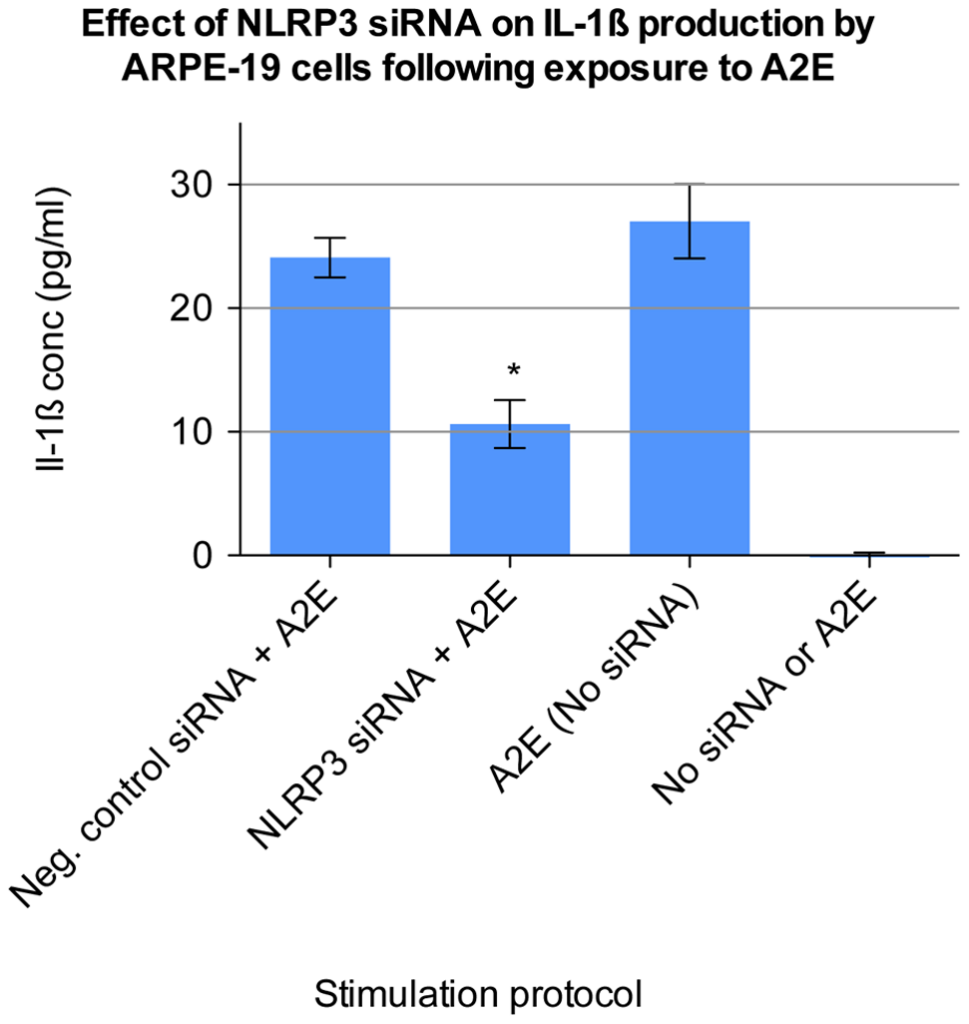
Effect of NLRP3 siRNA on IL-1ß production by ARPE-19 cells following exposure to A2E. Undifferentiated ARPE-19 cells were seeded onto 96-well plates and after 24 hours transfected for 72 hours with a 50 nM concentration of Silencer Select NLRP3 siRNA (Hs00918085_m1, Applied Biosystems Ltd). 50 nM of Silencer Select Negative Control No. 1 siRNA was also used. 1000 pg/ml Il-1α was added to the transfection media during this period. The cells were then stimulated for 24 hours with 10 µM A2E. Cell culture supernatant was collected and processed using ELISA. Cells were also incubated with transfection reagent alone, in the absence of any siRNA, and then stimulated with either A2E in DMEM, or DMEM alone. Eight separate wells were stimulated under each condition (n  = 8). Error bars represent standard deviation. (*) NLRP3 siRNA significantly inhibited IL-1ß production as compared to negative control siRNA (p<0.0001, one-way ANOVA).

NLRP3 siRNA significantly inhibited A2E induced IL-1ß production as compared to the negative control siRNA (p<0.0001, one-way ANOVA). The negative control siRNA had no significant effect upon IL-1ß production, as compared to incubation with transfection reagent alone (Figure 6).

### IL-1ß Release by A2E Stimulated ARPE-19 Cells was Dependent upon caspase-1

**Figure 7:**
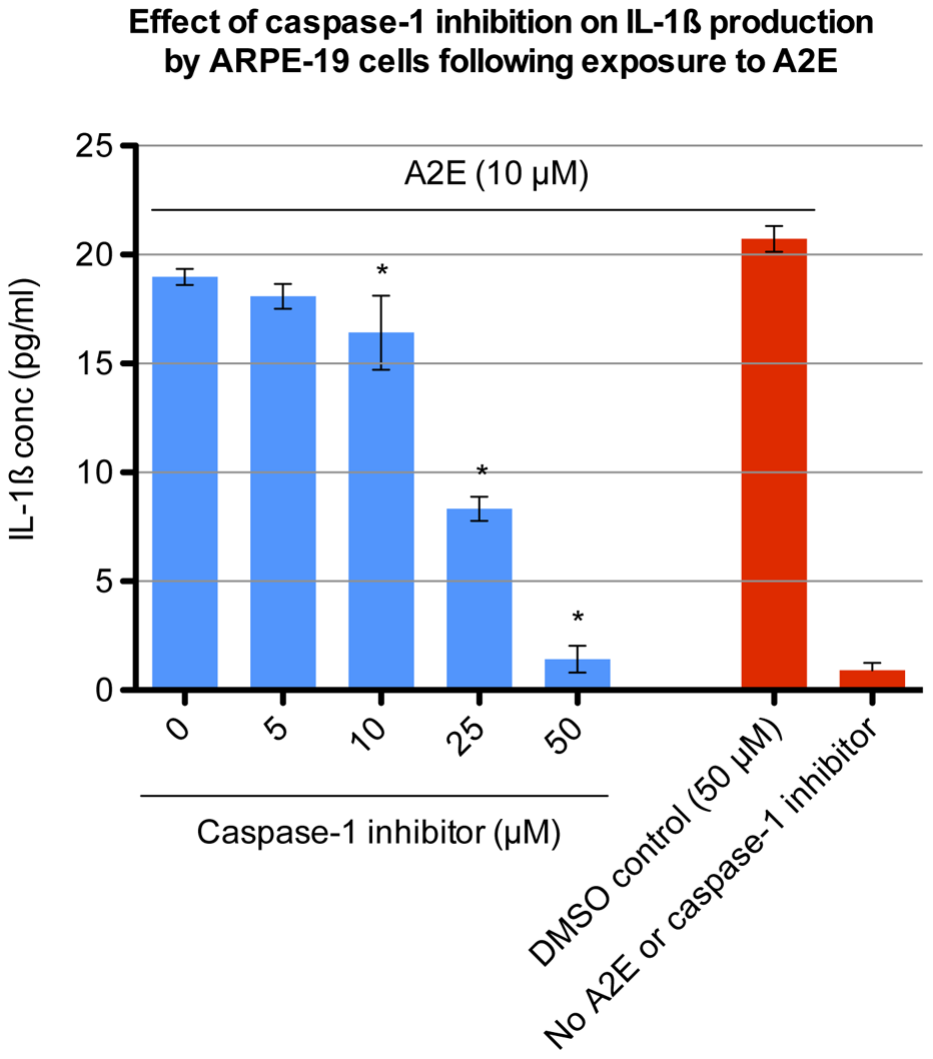
Effect of caspase-1 inhibition on IL-1ß production by ARPE-19 cells following exposure to A2E. Undifferentiated ARPE-19 cells were cultured in 96-well plates. The cells were pre-stimulated for 48 hours with 1000 pg/ml IL-1α and then incubated with 10 µM A2E for 24 hours in the presence of 0, 5, 10, 25, 50 µM of caspase-1 inhibitor. The caspase-1 inhibitor stock was dissolved in DMSO. Therefore DMSO at the same concentration, but without caspase-1 inhibitor, was used as a negative control. IL-1ß levels were recorded in the supernatant via ELISA. Four separate wells were stimulated with each concentration (n  = 4). Error bars represent standard deviation. (*) 10, 25 and 50 µM of caspase-1 inhibitor significantly inhibited IL-1ß production (p<0.0001, one-way ANOVA).

The NLRP3 inflammasome also contains activated caspase-1, directly converting pro-IL-1ß into mature IL-1ß. In order to confirm that A2E stimulates IL-1ß production through activation of caspase-1, the caspase-1 inhibitor Z-WEHD-FMK was used. The short peptide sequence (tryptophan-glutamic acid-histidine-aspartic acid) of this molecule covalently binds to activated caspase-1, irreversibly inhibiting its activity. Caspase-1 inhibition significantly inhibited A2E induced IL-1ß production, in a dose dependent manner (p<0.0001 for 10, 25 and 50 µM caspase-1 inhibitor, one-way ANOVA) (Figure 7). Interestingly, besides activation of IL-1ß, A2E also leads to RPE cell loss in the cultures [64], which we also observed. This cell loss was not rescued by the capase-1 inhibitor, suggesting that the cell death and IL-1ß release are distinct A2E-related events (Figure S4).

### ABCA4 Knockout Mice Demonstrate Increased Retinal Pigment Epithelial IL-1ß Staining

**Figure 8:**
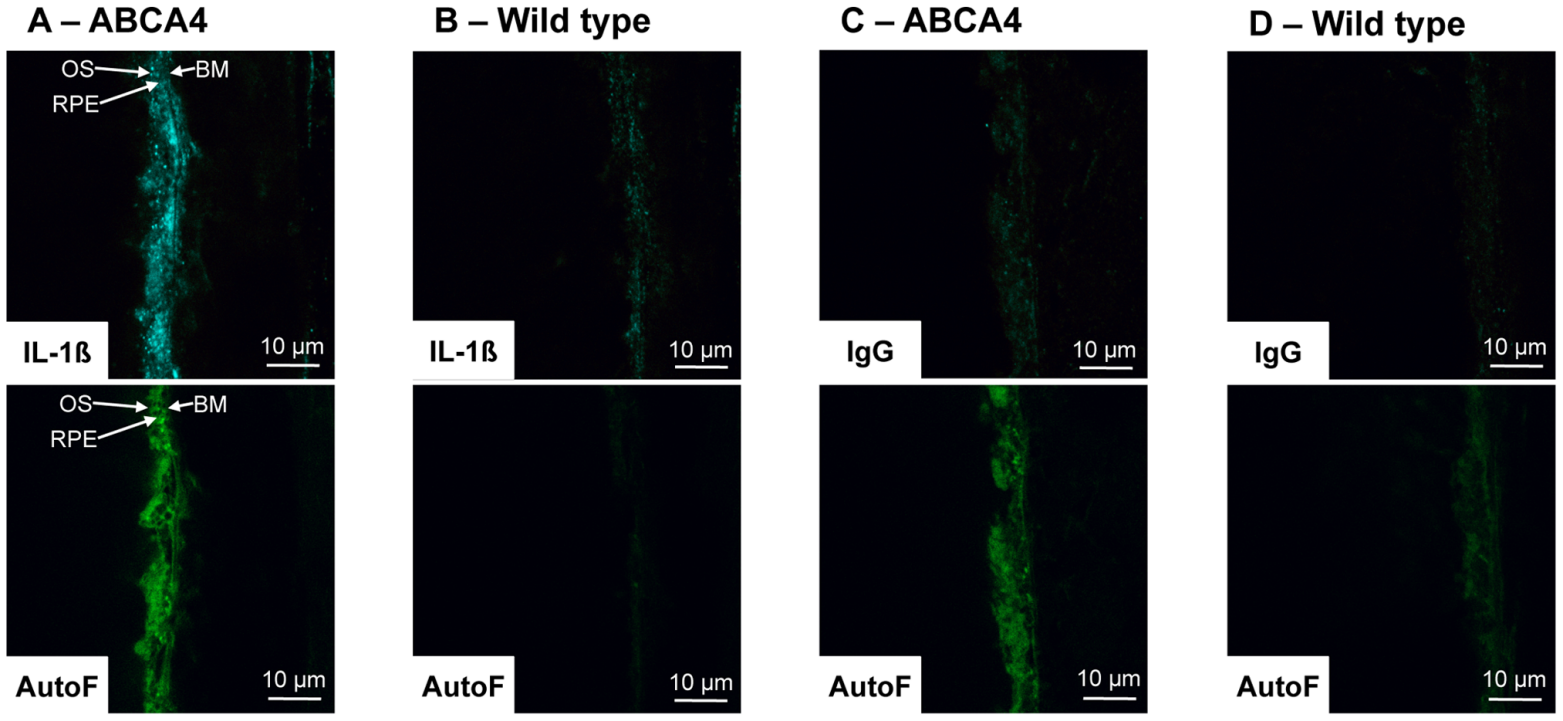
ABCA4 knockout mice demonstrate increased retinal pigment epithelial IL-1ß staining. Sections were stained for IL-1ß. Both matched wild type mice (129S2/SvHsd) and isotype matched IgG were used as negative controls. (**A & B**) Upper micrographs show IL-1ß staining of retinal pigment epithelium for ABCA4 knockout mice and SVHSD control mice. Increased IL-1ß staining is seen in the ABCA4 knockout mice compared to the wild type mice. Lower micrographs show RPE autofluorescence (AutoF - absorption 490 nm, emission 520 nm). Higher autoflourescence of the ABCA4 knockout mice is in keeping with increased levels of lipofuscin. (**C & D**) Upper micrographs show control IgG staining (primary antibody) of ABCA4 knockout mice and wild type mice. No significant staining is seen in both samples. Lower micrographs show RPE autofluorescence. Abbreviations: BM – Bruch’s membrene, OS – Outer segments, RPE – Retinal pigment epithelium.

Finally, one prediction that comes from our in vitro findings is that IL-1ß production should be elevated after exposure to A2E. To test this prediction, we chose to look at the ABCA4 knockout mouse. This mouse demonstrates increased levels of lipofuscin and A2E in the RPE with increasing age. [65]. Tissue sections were stained for IL-1ß and both age-matched wild type mice and isotype matched IgG were used as negative controls. ABCA4 knockout mice showed increased autofluoescence in the RPE, consistent with previous reports and indicative of accumulation of fluorophores such as A2E. A specific increase in IL-1ß staining was also observed in and around the RPE-photoreceptor outer segment junction in the ABCA4 knockout mouse, compared to the age-match controls (Figure 8).

## Discussion

In this study we demonstrated that RPE cells secrete a wide variety of chemokines and cytokines in response to stimulation with A2E. The other danger signals tested did not produce a significant rise in the tested chemokines or cytokines. Release of IL-1ß, in response to A2E, was dependent on PRR activation, in particular the NLRP3 inflammasome.

IL-1ß appears to play an early role in inflammatory chemokine/cytokine production by the RPE. Stimulation of RPE cells by IL-1ß has been shown to induce the production of IL-6, IL-8, and MCP-1 [53], [54], [55] (Table S1). Therefore activation of the PRR NLRP3, with subsequent IL-1ß production, appears to be an important initial step in the pathway of A2E induced chemokine/cytokine production by the RPE. Despite initial data suggesting endothelial cells do not respond similarly, it will be important to more thoroughly assess other relevant cell types for their cytokine response to A2E, such as photoreceptors and inflammatory cells such as macrophages. While it may be assumed that inflammatory chemokine production by RPE cells, in response to A2E is likely to be damaging, this may not be the case. The inflammatory cell recruitment may initially serve as a protective mechanism, enabling the scavenging of extracellular material and regulation of RPE lipofuscin/A2E content. Indeed impaired trafficking of leucocytes, in the MCP-1 (Ccl-2) double knockout model, leads to increased levels of A2E in the RPE. [3] A2E induced MCP-1 production may therefore initially serve as a regulatory, rather than pathological inflammatory mechanism. However with time this regulatory function may become pathological. Our in vivo data suggest that the ABCA4 mouse model of A2E accumulation may be very relevant for future exploration of the role of A2E-induced IL-1ß production, the inflammasome and RPE pathology akin to early changes in AMD and Stargardt’s.

The role of the NLRP3 inflammasome in the pathogenesis of AMD is a very new area of interest. NLRP3 activity has been demonstrated in a wide variety of other diseases, including Alzheimer’s disease, asbestosis and silicosis, gout, and atherosclerosis. [25], [60], [61], [62] It is notable that in all of these studies, the molecules which initiated NLRP3 inflammasome activity were all relatively insoluble (amyloid-ß, asbestos, silica, uric acid crystals, and cholesterol crystals). A2E, a major component of lipofuscin, is also insoluble under aqueous conditions. Ingested A2E collects in the ARPE-19 cells, forming visible intracellular deposits.

Several of our findings support the conclusion that the IL-1ß release was an active process, rather than due to cell damage, secondary to the oxidizing properties of A2E. First, the inhibitor of caspase-1 activity potently reduced IL-1ß release. In contrast, the caspase-1 inhibitor had no effect on A2E-induced cell loss, thus suggesting the two events are unrelated. Interestingly, Dynasore, the inhibitor of endocytosis, potently inhibited both IL-1ß release and the cell loss, suggesting that active internalization is required for at least these two bioactivities of A2E in ARPE-19.

The process of inflammasome activation in ARPE-19 was similar to that described for other insoluble NLRP3 inflammasome activators. The initial step involved ingestion, through endocytosis, followed by involvement of the inflammasome proteins ASC, NLRP3 and caspase-1. Our findings using the cathepsin-B inhibitor also suggest a role for this protease as a potential link between endocytosis and the inflammasome-based IL-1ß release. Lysosomal damage and release of cathepsins into the cytoplasm is proposed to trigger conversion of pro-caspase-1 to the active enzyme and activate the inflammasome. However CA-074 Me (used to inhibit cathepsin-B) has also been suggested to inhibit inflammasome activity through off-target effects. [66] Therefore the involvement of cathepsin-B requires further confirmation.

There have recently been some exciting discoveries concerning the role of the NLRP3 inflammasome in AMD. NLRP3 inflammasome activation and IL-18 secretion, involving peripheral blood mononuclear cells, has been shown to suppress the formation of laser induced choroidal neovascularisation (CNV), a model for the wet form of AMD. [22] Tarallo et al demonstrated increased levels of NLRP3, ASC, and caspase-1 in human eyes containing geographic atrophy. They also showed that inhibition of components of the NLRP3 inflammasome (NLRP3, ASC and caspase-1), in RPE, prevented RPE degeneration and atrophy induced by DICER1 loss or Alu RNA exposure. The pathway towards geographic atrophy, a key feature of dry AMD, involved MyD88 and IL-18. [23] Since A2E also activates the NLRP3 inflammasome, it will be interesting in the future to determine if A2E also has any direct effects on the processing of Alu RNA.

Amyloid-ß, CEP-HSA, CEP-Lysine, CML-HSA and vitronectin did not induce any chemokine/cytokine production in the RPE cells. Doyle et al found that stimulation with CEP modified proteins alone was not sufficient to activate NLRP3 inflammasome, in bone marrow derived macrophages. CEP modified proteins did have the ability to prime the NLRP3 inflammasome, enabling increased secretion of IL-1ß following stimulation with compounds such as ATP. [22] We also found that stimulation with CEP modified proteins alone was not sufficient to activate NLRP3 inflammasome, this time in a RPE cell model. CEP modified proteins are still likely to play an important role in promoting inflammation in AMD, through their priming effect upon the NLRP3 inflammasome.

This effect of A2E, in stimulating the production of chemokines and cytokines by RPE cells, has never been characterized before. A2E has been shown to promote complement activation, induced photo-oxidative cellular damage, and inhibit lysosomal protein degradation. [31], [45], [67], [68] All these effects have been implicated in the pathogenesis of AMD. The discovery that it also can induce chemokine and cytokine production by RPE cells, including IL-1ß production through activation of the NLRP3 inflammasome, further supports the link between A2E, inflammation and the pathogenesis of AMD. It also supports the recent discovery of NLRP3 inflammasome activation in AMD. A2E is an important component of lipofuscin, and appears not to be simply a byproduct of an aged retina. It’s potent inflammatory chemokine and cytokine induction capabilities make it a potential early contributor to inflammation in AMD.

## Supporting Information

Figure S1 **HPLC trace for crude A2E.** The two labeled peaks represent A2E and iso A2E respectively.
(TIFF)

Figure S2 **Six month old ARPE-19 cells show features of differentiation.** Transmission electron microscopy: (**A**) ARPE-19 cells form a monolayer with clear delineation between adjacent cells (arrows). Basal infoldings can also be seen (asterisk). (**B**) Microvilli are seen on the apical cell surface (arrow). Intra-cytoplasmic melanin granules in various stages of differentiation are also seen (asterisk) (**C**) Tight junctions are seen between adjacent cells at the apical side of the cell (arrow). Light microscopy: (**D**) ARPE-19 cells show multiple pigment granules consistent with intra-cytoplasmic melanin. Immunohistochemistry: (**E**) Six month old ARPE-19 cells on transwell inserts were stained with a 8 µg/ml rabbit anti-human zonula occludens-1 (Invitrogen Ltd) antibody followed by Alexa Fluor 488 goat anti-rabbit IgG (Invitrogen Ltd) at a 1∶200 dilution. Cells were mounted in Prolong Gold antifade reagent with DAPI.
(TIFF)

Figure S3 **IL-1ß production by differentiated ARPE-19 cells following exposure to A2E.** Undifferentiated ARPE-19 cells were treated with 0 and 20 µM A2E for a period of 24 hours and IL-1ß levels were recorded in the supernatant via ELISA. As A2E was dissolved in DMSO, cells were also stimulated with DMSO only, to exclude an effect from DMSO. Six separate wells were stimulated with each concentration (n  = 6). Error bars represent standard deviation. (*) 20 µM A2E significantly increased IL-1ß production (p<0.0001, one-way ANOVA).
(TIFF)

Figure S4 **Effect of A2E on cell survival over 24 hours.** Undifferentiated ARPE-19 cells were incubated with either 0 or 10 µM A2E for 24 hours. The supernatant was then removed and the adherent cells trypsinised, resuspended in tryphan blue, and counted using a haemocytometer. Cells were also incubated in the presence of both 10 µM A2E and 50 µM caspase-1 inhibitor. Eight separate wells were stimulated with each method (n  = 8) (*) 10 µM A2E significantly reduced the number of viable adherent ARPE-19 cells (p<0.0001, unpaired t test). This reduction in cells was not rescued by the presence of the caspase-1 inhibitor.
(TIFF)

Text S1 **Supporting Methods.**
(DOCX)

Table S1 **Multiplex assay of cell culture supernatant from ARPE-19 cells stimulated with IL-1α.**
(DOCX)
