# Supplementary figures and images for: A2E Induces IL-1ß Production in Retinal Pigment Epithelial Cells via the NLRP3 Inflammasome
Source: PLoS One. 2013 Jun 28;8(6):e67263. doi: 10.1371/journal.pone.0067263 (PMC3696103; doi:10.1371/journal.pone.0067263)

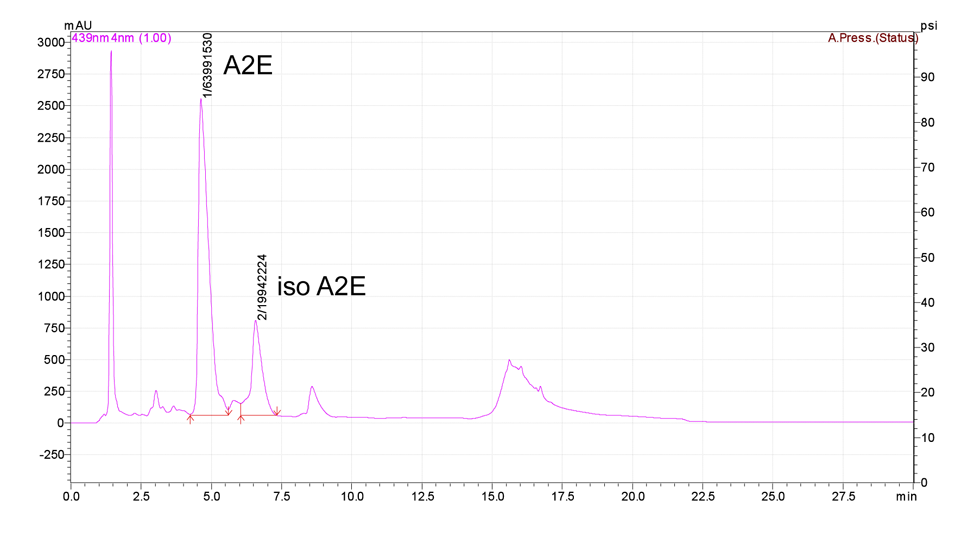

Supplement: Figure S1 — HPLC trace for crude A2E. The two labeled peaks represent A2E and iso A2E respectively. (TIFF) [file pone.0067263.s001.tiff]

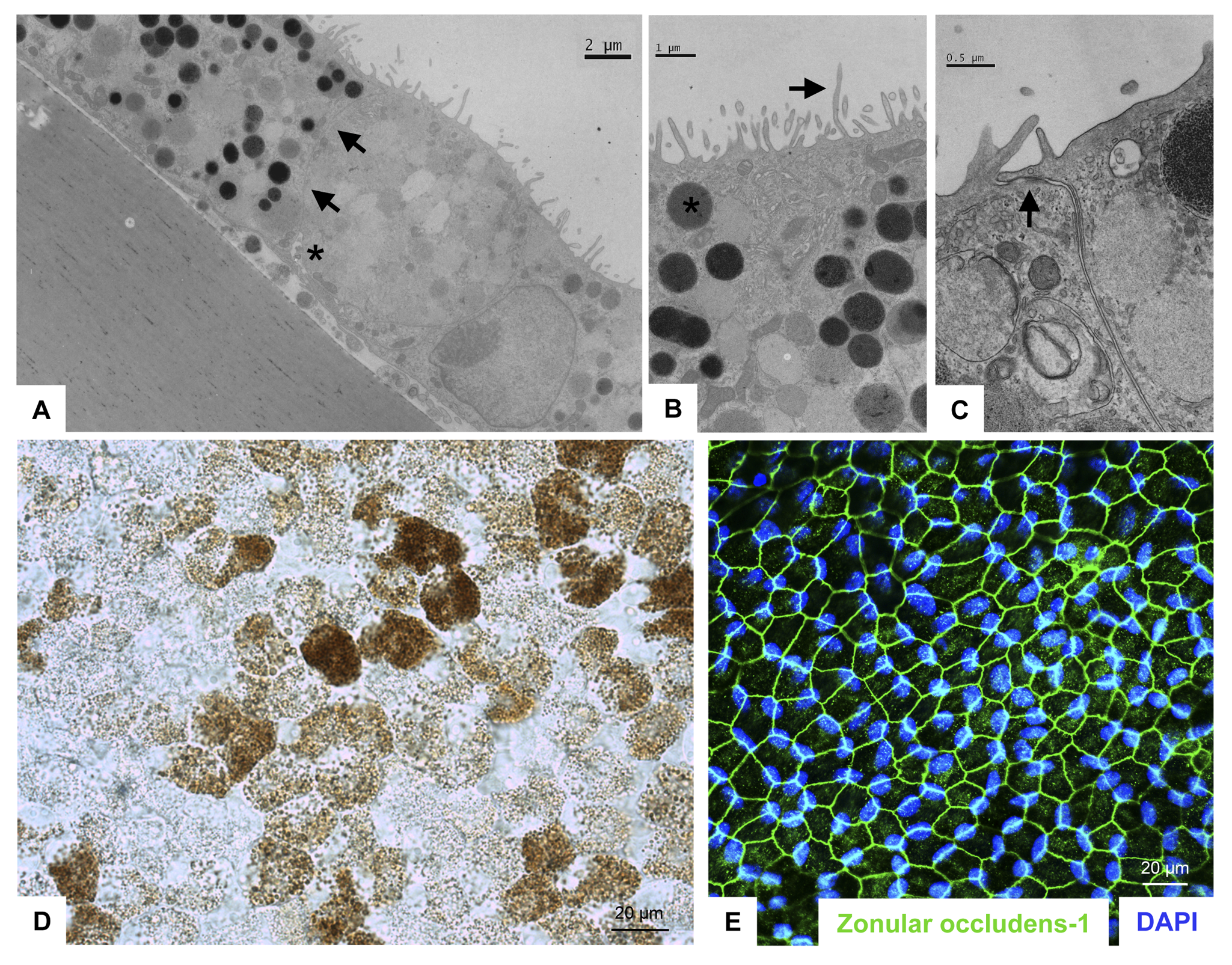

Supplement: Figure S2 — Six month old ARPE-19 cells show features of differentiation. Transmission electron microscopy: (A) ARPE-19 cells form a monolayer with clear delineation between adjacent cells (arrows). Basal infoldings can also be seen (asterisk). (B) Microvilli are seen on the apical cell surface (arrow). Intra-cytoplasmic melanin granules in various stages of differentiation are also seen (asterisk) (C) Tight junctions are seen between adjacent cells at the apical side of the cell (arrow). Light microscopy: (D) ARPE-19 cells show multiple pigment granules consistent with intra-cytoplasmic melanin. Immunohistochemistry: (E) Six month old ARPE-19 cells on transwell inserts were stained with a 8 µg/ml rabbit anti-human zonula occludens-1 (Invitrogen Ltd) antibody followed by Alexa Fluor 488 goat anti-rabbit IgG (Invitrogen Ltd) at a 1∶200 dilution. Cells were mounted in Prolong Gold antifade reagent with DAPI. (TIFF) [file pone.0067263.s002.tiff]

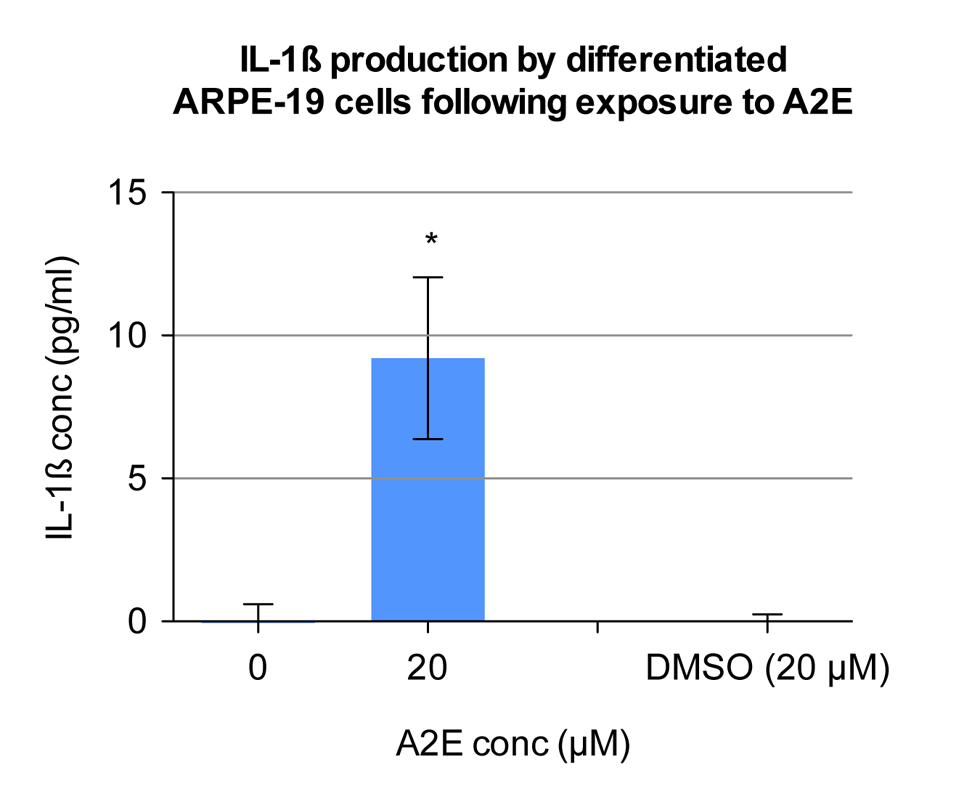

Supplement: Figure S3 — IL-1ß production by differentiated ARPE-19 cells following exposure to A2E. Undifferentiated ARPE-19 cells were treated with 0 and 20 µM A2E for a period of 24 hours and IL-1ß levels were recorded in the supernatant via ELISA. As A2E was dissolved in DMSO, cells were also stimulated with DMSO only, to exclude an effect from DMSO. Six separate wells were stimulated with each concentration (n = 6). Error bars represent standard deviation. (*) 20 µM A2E significantly increased IL-1ß production (p<0.0001, one-way ANOVA). (TIFF) [file pone.0067263.s003.tiff]

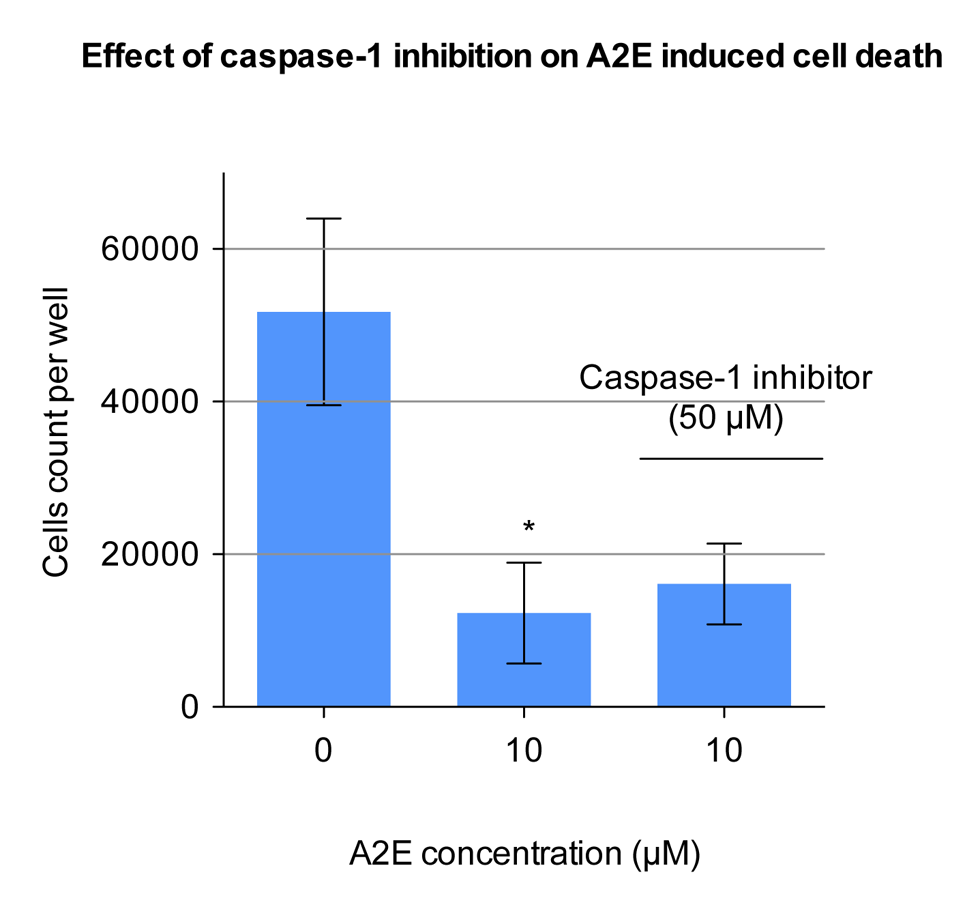

Supplement: Figure S4 — Effect of A2E on cell survival over 24 hours. Undifferentiated ARPE-19 cells were incubated with either 0 or 10 µM A2E for 24 hours. The supernatant was then removed and the adherent cells trypsinised, resuspended in tryphan blue, and counted using a haemocytometer. Cells were also incubated in the presence of both 10 µM A2E and 50 µM caspase-1 inhibitor. Eight separate wells were stimulated with each method (n = 8) (*) 10 µM A2E significantly reduced the number of viable adherent ARPE-19 cells (p<0.0001, unpaired t test). This reduction in cells was not rescued by the presence of the caspase-1 inhibitor. (TIFF) [file pone.0067263.s004.tiff]
