# Supplementary material for: A2E Induces IL-1ß Production in Retinal Pigment Epithelial Cells via the NLRP3 Inflammasome
Source: PLoS One. 2013 Jun 28;8(6):e67263. doi: 10.1371/journal.pone.0067263 (PMC3696103; doi:10.1371/journal.pone.0067263)
Supplement: Text S1 — Supporting Methods. (DOCX) [file pone.0067263.s005.docx]

**Supporting methods**

**Differentiated cell culture.** Transwell polyester membrane inserts, pore diameter 0.4 μm, membrane diameter 12 mm (Corning Ltd) were used for long term cultures. The upper (cell adhesion) surface of the membrane was coated with 240 µl of 8 µg/ml laminin in PBS for 2 hours at 37^o^C. Each well was then washed three times with 500 µl PBS and stored in PBS while awaiting seeding of cells. The upper chamber of each well was seeded with 100 000 cells (in 500 µl DMEM/F12 containing 10% FBS, 100 units/ml penicillin, 100 μg/ml streptomycin). 1.5 ml of DMEM/F12 containing 10% FBS, 100 units/ml penicillin, 100 μg/ml streptomycin was placed into the lower chamber. The Transwell plates were placed into an incubator at 37^o^C in 5% CO_2_.

After 24 hours the medium on both sides of the membrane was replaced with pyruvate-free D-MEM (4500 mg/L D-glucose, 4 mM L-Glutamine, no pyruvate, Invitrogen Ltd) supplemented with 1% FBS, 100 units/ml penicillin, 100 μg/ml streptomycin. Cell culture medium was changed twice weekly. Care was taken not to damage the cell layer during this process.

In order to prepare cells for cytokine release differentiated cells were starved for 24 hours, then prestimulated with 1000 pg/ml recombinant human IL-1α for 48 hours (delivered to both sides of the membrane). The medium used in each step was the same as for undifferentiated cells. However the volumes were different with 500 µl placed in the top chamber and 1500 µl in the bottom chamber. 20 µM A2E, in 500 µl serum free DMEM (pyruvate-free DMEM containing 100 units/ml penicillin, 100 μg/ml streptomycin only) was then applied to the upper chamber containing the cells. 500 µl of serum free DMEM (without A2E) was applied to the lower chamber. The cells were incubated for 24 hours. Following this period of stimulation the Transwell plates were centrifuged at 800 rpm for 10 minutes and the top 150 µl of the supernatant was removed from each chamber containing the cells. This was frozen at -80^o^C for later processing by ELISA.

**Assessment of purity and molecular weight of A2E.** Purity of A2E fractions was assessed using high performance liquid chromatography (HPLC). Molecular weight was assessed using matrix-assisted laser desorption/ionization mass spectrometry (MALDI-MS). An Axima-CFR MALDI (Shimazu Ltd) was used. Samples were placed on a stainless steel MALDI plate and co-crystalised with α-cyano-4-hydroxy-cinnamic acid MALDI matrix (Thermo Scientific Ltd). A UV nitrogen laser (337 nm) was applied, and the mass of the resultant ions was recorded. The MALDI-MS was calibrated against bradykinin fragment 1-7 (756.4 Da), human angiotensin-2 (1046.2 Da) and the MALDI matrix material (188.2 Da), in order that accurate molecular weight recordings could be achieved.

We were satisfied as to the quality of manufactured A2E. Following HPLC purification of crude A2E, two peaks were seen at between 4 and 7 minutes into the run (Figure S1). This set of peaks had the same configuration to the peaks of A2E and iso-A2E, reported in the literature. [1-6] The ratio of areas under the peaks was 3.2:1 (A2E : iso A2E). This was similar to the 4:1 ratio reported in the literature, regarding the equilibrium between A2E and iso A2E under ambient lighting conditions. [3] The purity of the collected A2E peak, assessed using HPLC, was 99.4%. The molecular weight of this peak was assessed using MALDI mass spectrometry. The m/z (mass/charge) value obtained, for a single charged positive ion, was 592.5. This was comparable to the m/z values reported in the literature for A2E. Jang et al and Wang et al reported an m/z value of 592, while Gutierrez et al reported a value of 592.5 [2,7,8]

**Assessment of concentration and modification of CEP modified human serum albumin.** The final protein concentration was determined using the Pierce bicinchoninic acid (BCA) protein assay (Thermo Scientific Ltd) using a bovine serum albumin (BSA) standard curve. To perform this assay 1 ml of BCA working reagent reagent (50 parts BCA reagent A and 1 part BCA reagent B) was mixed with 50 µl protein solution (in PBS). This mixture was incubated at 37^o^C for 30 minutes, and then returned quickly to room temperature. Absorbance was measured at 562 nm and compared with a standard curve prepared with bovine serum albumin.

The effectiveness of DBU at removing Fm groups was assayed using UV spectroscopy, (monitoring absorption at 265 nm). The pyrrole concentration was determined by adding 4-(dimethylamino)benzaldehyde (DMAB, Ehrlich reagent). This generates a characteristic chromophore. To perform this assay 100 ul of CEP-HSA, in PBS, was added to 1.3 ml of 1% (wt/vol) sodium dodecyl sulfate (SDS) in water. 100 ul of Ehrlich reagent was then added. This contained 1.5% (wt/vol) DMAB and 1% hydrochloric acid (vol/vol) in 50:50 methanol:water. After 10 minutes incubation at room temperature, the absorbance was measured at 530 nm. 2,5-dimethylpyrrole was used as a quantitative standard. [9]

The concentration of CEP-HSA (BCA protein assay) was 1.62 mg/ml (24.33 µM using the molecular weight of HSA). The pyrrole concentration in the newly prepared CEP-HSA was 105.2 µM. With a CEP-HSA concentration of 24.33 µM and a pyrrole concentration of 105.2 µM, each CEP-HSA molecule contained an average of 4.33 pyrrole groups. Unmodified HSA was also assessed. No pyrrole modifications were found on unmodified HSA. All assays were performed in triplicate.

**Supporting results**

**Long-term ARPE-19 cultures, on Transwell inserts, produced pigmented cells with many of the characteristics of differentiated retinal pigment epithelium**

A2E induced the production of IL-1ß by undifferentiated ARPE-19 cells. To assess whether this still occurred in cells showing features more like differentiated retinal pigment epithelium, ARPE-19 cells were cultured to promote differentiation.

ARPE-19 cells were cultured on Transwell polyester membrane inserts. After 3 months they began to become pigmented. By 6 months pigment granules were clearly visible. Transmission electron microscopy of 6-month old cells demonstrated many features seen in differentiated retinal pigment epithelial cells. The cells formed monolayers with clearly defined cell borders. Intra-cytoplasmic melanin granules, in various stages of maturation, were seen. The cells demonstrated polarized features, such as apical microvilli and apical tight junctions. In addition immunocytochemistry demonstrated an intact network of zonula occludens-1 staining between adjacent cells, further confirming the presence of tight junctions (Figure S2). These differentiated ARPE-19 cells also showed some basolateral membrane infoldings.

**Differentiated ARPE-19 cultures also secrete IL-1ß in the presence of A2E**

Differentiated ARPE-19 cells were pre-stimulated for 48 hours with 1000 pg/ml IL-1α. Cells were then incubated with 0 and 20 µM of A2E for 24 hours and cell culture supernatant was collected and processed using ELISA. A2E stock was dissolved in DMSO. Therefore DMSO at the same concentration, but without A2E, was used as a negative control. Six separate wells were stimulated with each concentration (n = 6). Stimulation with 20 µm A2E induced a significant increase in IL-1ß production (p<0.05) (Figure S3).

**Supporting references**

1. Bhosale P, Serban B, Bernstein PS (2009) Retinal carotenoids can attenuate formation of A2E in the retinal pigment epithelium. Arch Biochem Biophys 483: 175-181.

2. Gutierrez DB, Blakeley L, Goletz PW, Schey KL, Hanneken A, et al. (2010) Mass spectrometry provides accurate and sensitive quantitation of A2E. Photochem Photobiol Sci 9: 1513-1519.

3. Parish CA, Hashimoto M, Nakanishi K, Dillon J, Sparrow J (1998) Isolation and one-step preparation of A2E and iso-A2E, fluorophores from human retinal pigment epithelium. Proc Natl Acad Sci U S A 95: 14609-14613.

4. Sparrow JR, Kim SR, Wu Y (2010) Experimental approaches to the study of A2E, a bisretinoid lipofuscin chromophore of retinal pigment epithelium. Methods Mol Biol 652: 315-327.

5. Wu Y, Zhou J, Fishkin N, Rittmann BE, Sparrow JR (2011) Enzymatic degradation of A2E, a retinal pigment epithelial lipofuscin bisretinoid. J Am Chem Soc 133: 849-857.

6. Zhou J, Jang YP, Kim SR, Sparrow JR (2006) Complement activation by photooxidation products of A2E, a lipofuscin constituent of the retinal pigment epithelium. Proc Natl Acad Sci U S A 103: 16182-16187.

7. Jang YP, Matsuda H, Itagaki Y, Nakanishi K, Sparrow JR (2005) Characterization of peroxy-A2E and furan-A2E photooxidation products and detection in human and mouse retinal pigment epithelial cell lipofuscin. J Biol Chem 280: 39732-39739.

8. Wang Z, Keller LM, Dillon J, Gaillard ER (2006) Oxidation of A2E results in the formation of highly reactive aldehydes and ketones. Photochem Photobiol 82: 1251-1257.

9. DeCaprio AP, Jackowski SJ, Regan KA (1987) Mechanism of formation and quantitation of imines, pyrroles, and stable nonpyrrole adducts in 2,5-hexanedione-treated protein. Mol Pharmacol 32: 542-548.
