# Supplementary material for: A2E Induces IL-1ß Production in Retinal Pigment Epithelial Cells via the NLRP3 Inflammasome
Source: PLoS One. 2013 Jun 28;8(6):e67263. doi: 10.1371/journal.pone.0067263 (PMC3696103; doi:10.1371/journal.pone.0067263)
Supplement: Table S1 — Multiplex assay of cell culture supernatant from ARPE-19 cells stimulated with IL-1α. (DOCX) [file pone.0067263.s006.docx]

**Table S1. Multiplex assay of cell culture supernatant from ARPE-19 cells stimulated with IL-1α.**

|  |  | Nil | IL-1α | Stat. signif. |
| --- | --- | --- | --- | --- |
| Chemokines | IL-8 | 201.1 (21.5) | 1959.0 (94.7) | **Yes** |
|  | MCP-1 | 2966.0 (237.2) | 8851.3 (131.2) | **Yes** |
|  | MIG | 0.0 (0.0) | 0.0 (0.0) | No |
|  | MIP-1α | 0.0 (0.0) | 0.0 (0.0) | No |
|  | MIP-1ß | 0.0 (0.0) | 0.0 (0.0) | No |
| Cytokines | G-CSF | 0.0 (0.0) | 0.0 (0.0) | No |
|  | IFN-γ | 0.0 (0.0) | 0.0 (0.0) | No |
|  | IL-1ß | 0.0 (0.0) | 0.0 (0.0) | No |
|  | IL-2 | 0.0 (0.0) | 0.0 (0.0) | No |
|  | IL-6 | 0.0 (0.0) | 198.0 (16.2) | **Yes** |
|  | TNF-α | 0.0 (0.0) | 0.0 (0.0) | No |
| Other | VEGF-A | 746.0 (58.4) | 1242.6 (21.4) | **Yes** |

Cells were pre-stimulated with for 48 hours with or without (‘Nil’ - negative control) 1000 pg/ml IL-1α (IL-1 receptor agonist). They were then incubated for 24 hours with DMEM only. Cell culture supernatant was then assessed for the presence of twelve different proteins (grouped as chemokines, cytokines and other). Concentrations of these proteins are expressed in pg/ml. Mean values (with standard deviation in brackets) are presented. Each assay was performed thee times (n = 3) using three separate cell culture wells.

DMEM = Dulbecco's Modified Eagle Medium, Stat. signif. = Statistical significance
